# Cytotoxic and Antitumor Activity of Lactaptin in Combination with Autophagy Inducers and Inhibitors

**DOI:** 10.1155/2019/4087160

**Published:** 2019-06-17

**Authors:** Anastasia V. Bagamanshina, Olga S. Troitskaya, Anna A. Nushtaeva, Anastasia Yu Yunusova, Marina O. Starykovych, Elena V. Kuligina, Yuri Ya Kit, Max Richter, Fabian Wohlfromm, Thilo Kähne, Inna N. Lavrik, Vladimir A. Richter, Olga A. Koval

**Affiliations:** ^1^Institute of Chemical Biology and Fundamental Medicine, Siberian Branch, Russian Academy of Sciences, Lavrentiev Ave. 8, 630090 Novosibirsk, Russia; ^2^Institute of Cell Biology, Dragomanova Str. 16, 79000 L'viv, Ukraine; ^3^Otto von Guericke University, Universitätspl. 2, 39106 Magdeburg, Germany; ^4^Novosibirsk State University, Pirogova Str. 1, 630090 Novosibirsk, Russia

## Abstract

Autophagy is a degradative process in which cellular organelles and proteins are recycled to restore homeostasis and cellular metabolism. Autophagy can be either a prosurvival or a prodeath process and remains one of the most fundamental processes for cell vitality. Thus autophagy modulation is an important approach for reinforcement anticancer therapeutics. Earlier we have demonstrated that recombinant analog of human milk protein lactaptin (RL2) induced apoptosis of various cultured cancer cells and activated lipidation of microtubule-associated protein 1 light chain 3 (LC3). In this study we investigated whether autophagy inhibitors—chloroquine (CQ), Ku55933 (Ku), and 3-methyladenine (3MA)—or inducer—rapamycin (Rap)—can enhance cytotoxic activity of lactaptin analog in cancer cells and its anticancer activity in the mice model. Western Blot analysis revealed that RL2 induced short-term autophagy in MDA-MB-231 and MCF-7 cells at early stages of incubation and that these data were confirmed by the transmission electron microscopy of autophagosome/autophagolysosome formation. RL2 stimulates reactive oxygen species (ROS) production, autophagosomes accumulation, upregulation of ATG5 with processing of LC3I to LC3II, and downregulation of p62/sequestosome 1 (p62). We have shown that autophagy modulators, CQ, Ku, and Rap, synergistically increased cytotoxicity of RL2, and RL2 with CQ induced autophagic cell death. In addition, CQ, Ku, and Rap in combination with RL2 decreased activity of lysosomal protease Cathepsin D. More importantly, combining RL2 with CQ, we improved antitumor effect in mice. Detected synergistic cytotoxic effects of both types of autophagy regulators, inhibitors, and inducers with RL2 against cancer cells allow us to believe that these combinations can be a basis for the new anticancer approach. Finally, we suppose that CQ and Rap promoting of short-term RL2-induced autophagy interlinks with final autophagic cell death.

## 1. Introduction

Autophagy is a cellular process, which is essential for all multicellular organisms. When autophagy is initiated, cellular organelles and proteins are engulfed by autophagosomes, digested in autophagolysosomes, and recycled to restore homeostasis and cellular metabolism. There is no doubt that targeting autophagy is a very promising strategy for the treatment of various diseases, including cancer [1–7]. In cancer biology autophagy usually promotes tumor progression as being one of the fundamental mechanisms which allows tumors to survive in nutrient-deprived or hypoxic conditions [8, 9]. Moreover, anticancer drugs can also activate autophagy in cancer cells, which results in the decrease of efficiency of chemotherapeutics [7, 10, 11]. For convenient anticancer chemotherapeutics such as doxorubicin, cisplatin, and methotrexate [8], activation of prosurvival autophagy has already been demonstrated. But in some cases autophagy accelerates cell death and can stimulate tumor suppression [12]. Therefore, correct regulation of autophagy is an important antineoplastic strategy [9].

Earlier we showed that recombinant analog of lactaptin RL2 suppresses tumor growth and metastasis in mice with no signs of toxic effects [13]. Besides apoptosis, RL2 induced processing of microtubule-associated protein 1 light chain 3 (LC3) which is referred to as a marker of autophagy. When RL2 was used* in vitro* in MDA-MB-231 cells with autophagy inhibitor chloroquine, this combination was more cytotoxic than RL2 or CQ alone [14]. Therefore, we supposed that treatment of lactaptin analog with various autophagy inducers or inhibitors has the potential for improving of cytotoxic and anticancer effect of RL2.

**Figure 1:**
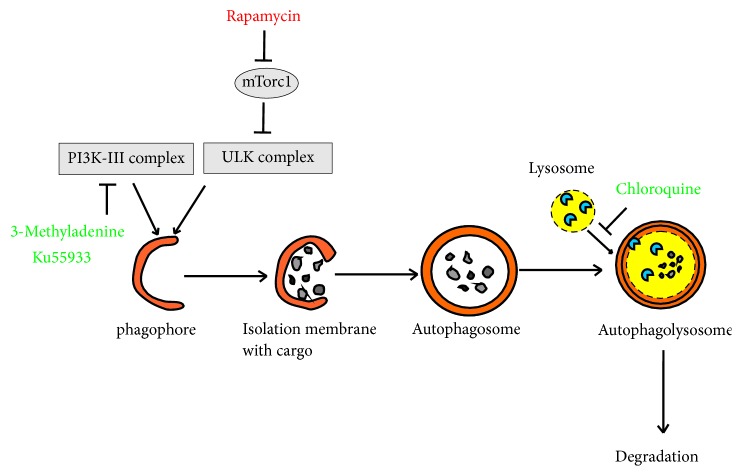
Key points of autophagy modulation by various drugs.

In this study we used a set of various autophagy inhibitors and inducers which switch over diverse steps in autophagy pathway (see Figure 1). 3-Methyladenine (3MA) is a widely used inhibitor of autophagy which suppresses phosphoinositide-3-kinases (PI3Ks) activity [15, 16] leading to suppression of autophagosome formation [17]. Chloroquine prevents fusion of autophagosomes with lysosomes [16, 18], while Ku55933 (Ku), an ATM kinase inhibitor [19], acts like 3MA by blocking class III PI3K [20]. Spermidine induces macroautophagy by inhibiting the acetyltransferase EP300 [21]. Rapamycin activates autophagy inhibiting mTOR signaling pathway [22].

Here we tried to reveal which autophagy inhibitor or inducer enhances cytotoxic activity of lactaptin analog RL2* in vitro* and* in vivo* with the highest degree and to discover activated death pathways by these combinations of compounds.

## 2. Experimental Section

### 2.1. Materials

#### 2.1.1. Cell Lines and Mice

MCF-7 human breast adenocarcinoma cells and MDA-MB-231 human breast adenocarcinoma cells were obtained from the Russian cell culture collection (Russian Branch of the ETCS, St. Petersburg, Russia). The RLS murine lymphosarcoma cells were generously provided by Dr. V. I. Kaledin (Institute of Cytology and Genetics SB RAS, Novosibirsk, Russia). Cells were maintained as previously published [23].

Female CBA mice (8-9 weeks old) were obtained from SPF vivarium of the Institute of Cytology and Genetics SB RAS, Novosibirsk, Russia.

#### 2.1.2. Antibodies and Chemicals

The following antibodies and chemicals were obtained from commercial sources: anti-LC3B and anti-ATG5 (Abcam, USA), anti-p62/SQSTM (Sigma-Aldrich, USA), polyclonal rabbit-anti-mouse and mouse-anti-rabbit HRP-conjugated secondary antibodies (Biosan, Russia), trypsin (Gibco, USA), inhibitor of trypsin from soybean (Paneco, USA), FITC Annexin V Apoptosis detection kit (BD Pharmingen, USA), and Ku55933 (Ku) (Tocris Bioscience, UK). Chloroquine, 3-methyladenine, rapamycin, and spermidine were purchased from Sigma-Aldrich, USA.

### 2.2. Methods

#### 2.2.1. MTT Assay

Cells were seeded in 96-well plates at 2 × 10^3^ cells per well in a volume of 100 *μ*L and cultivated under standard conditions in RPMI medium with 10% FBS, 0.3 mg/mL L-glutamine, 100 *μ*g/mL streptomycin, and 100 U/mL penicillin. After 24 hours, the cells were added to 100 *μ*L of test compounds dissolved in RPMI medium. After 48 hours, the medium was replaced with 200 *μ*L of serum-free RPMI medium with MTT (0.25 mg/mL) and cell were incubated for 4 hours at 37°C. Finally, the medium was removed from wells and formazan crystals were dissolved in DMSO. The optical density of the solution was measured at *λ* = 570 nm using a multichannel spectrophotometer. The effect of combination treatment was calculated with CompuSyn soft version 1.0. The MTT assay data of monotreatment and combination treatment were used for the calculation of CI by the software. Synergy (CI<1) or additivity (CI=1) effects were revealed according to the Chou-Talalay range.

#### 2.2.2. Reactive Oxygen Species (ROS) Measurement

For ROS measurement, two fluorescent probes were used: dihydroethidium (DHE, O_2_^−^-specific probe) and 2′,7′-dichlorofluorescin diacetate (DCFDA, detecting mainly H_2_O_2_). ROS content was measured in control (untreated) or RL2-treated (for 6, 12, and 24 h) cells after they were stained by fluorescent probes (10 *μ*M) for 30 min at 37°C. using FACScan flow cytometer (BD Biosciences, Mountain View, CA). All experiments were repeated three times with three parallel repeats. The Analysis of Variance (ANOVA) was used as a statistical test for the comparison of the experimental groups. Two-way ANOVA with Bonferroni posttests in order to compare replicated means by rows using GraphPad Prism v6.0 software was applied.

For ROS visualization, MDA-MD-231 cells were grown on glass slides, treated with RL2 or PBS (control), and finally incubated with 10 *μ*M DCFDA for 30 min at 37°C. After incubation, cells were immediately analyzed by fluorescence microscope Zeiss Axio Imager A1 (Carl Zeiss, Germany) at wavelength Ex/Em= 495/529 nm.

#### 2.2.3. Western Blot Analysis

Cells were lysed in lysis buffer (20 mM Tris, 150 mM NaCl, 1 mM EDTA, 1% NP40, pH 7.5). Aliquots of cell lysates were analyzed by 15% SDS-PAGE in a buffer for 50 mM Tris-HCl, 100 mM *β*-mercaptoethanol, 1% SDS, 10% glycerol, bromophenol blue (75 ng/mL), and xylene cyanol (75 ng/mL, pH 6,8) and transferred to a Trance-blot nitrocellulose membrane (Bio-Rad Laboratories, USA) by a wet blotting procedure (150V, 1 h, 10°C) in NuPAGE buffer (Novex Life Technologies, USA). The nitrocellulose membrane was processed by buffer iBind Solution Kit (Novex Life Technologies, USA); then the membrane was incubated in iBind Western apparatus (Life Technologies, USA) with primary antibodies and HRP-conjugated secondary antibodies for 18h at +4°C. Visualization of bound antibodies was achieved using a chemiluminescent substrate for HRP (Invitrogen, USA) on a C-Digit device (LI-COR, USA) with a software package, Image Studio Ver 4.0 C-DiGit.

#### 2.2.4. Apoptosis Detection

MDA-MB-231 and MCF-7 cells were seeded in 24-well plates and cultivated under standard conditions. When cells grew up to 80% monolayer, they were treated with tested compounds for various time intervals. Before trypsinization, detached cells were collected. Adhesive cells were rinsed with PBS and detached from the plate with trypsin. Trypsin inhibitor from soybean was used to inhibit trypsin-initiated proteolysis. Detached and trypsinized cells were combined, and then they were centrifuged for 5 min (0.8×10^3^ rpm). To detect cell death, FITC Annexin V Apoptosis detection kit and FACSCanto II (BD Biosciences, San Jose, CA) flow cytometer were used.

#### 2.2.5. Transmission Electron Microscopy

MDA-MB-231 cells were seeded in 6-well plates at 10^5^ cells per well and cultured under standard conditions. After 24 hours, drugs were added to the cells. After 3.5 hours of incubation at + 37°C, 100 *μ*L of FBS was added to the cells and the incubation was continued. After 4, 8, and 24 hours, cells were detached with 0.5% trypsin, diluted by L-15 medium (1 mL) with 10% FBS, and centrifuged for 5 min at 3000 rpm. Precipitated cells were fixed with 4% paraformaldehyde. Cell pellets were extra fixed with 1% osmium tetroxide solution for 2 h and then they were sequentially dehydrated in ethanol solutions rising concentration from 30% to 96%. The precipitates were washed with absolute acetone solution 5 times for 15 min and then they were heated in a mixture of resin (Araldite 502:SPI-PON 812: DDSA in a ratio of 1:1.5:3.7) and acetone (1:1) for 24 h. Air-dried cell pellets were placed in the conical molds and poured with resin mixed with catalyst (DMP-30). Obtained blocks were cut on ultramicrotome EM UC7 (Leica, Germany); the sections were contrasted with uranyl acetate and plumbum citrate solutions. Contrast ultrathin sections were examined in a transmission electron microscope JEM 1400 (JEOL, Japan) equipped with a Veleta digital camera (Olympus Soft Imaging Solution, Germany).

#### 2.2.6. External ATP Assay

Cells were seeded in 24-wells plates in IMDM medium without a phenol indicator with 10% FBS, 2 mM L-glutamine, and antimycotic antibiotic solution. The next day, cells were treated with drugs, and after the end of incubation the medium was collected. Extracellular ATP was measured in the conditioned media using ENLITEN ATP Assay System Bioluminescence Detection kit (Promega, USA), based on luciferin-luciferase conversion according to the following reaction:

ATP+D-Luciferin+O_2_→Oxyluciferin+AMP+PPi+CO_2_+Light

For the measurement of the light intensity (560 nm), a luminometer CLARIOstar (BMG Labtech, Germany) was used.

#### 2.2.7. Cathepsin D Activity

Cells (2x10^5^) were plated in six-well plates, treated with investigated compounds for 24 h and 48 h. Cathepsin D activity in the cell lysates was assayed using a Cathepsin D activity assay kit (Abcam, England, # ab65302). The kit is a fluorescent-based assay that utilizes the preferred Cathepsin D substrate sequence GKPILFFRLK(Dnp)-D-R-NH2) labeled with methyl coumaryl amide (MCA). A sample preparation was made according to the manufacturer's instructions. The resulting mixtures were analyzed using a fluorimeter (Cary Eclipse, Varian, Australia) at Ex/Em=328/400 nm using 5 nm excitation and emission slits.

#### 2.2.8. Protein Immunoprecipitation and Mass Spectrometry Analysis

The analysis of RL2-binding partners was performed by *κ*-Casein immunoprecipitation. The *κ*-Casein antibody (Anti-CSN3 antibody, ab111406, Abcam) was covalently coupled to sepharose beads using Pierce™ Co-Immunoprecipitation Kit according to manufacturer's instructions (Thermo Fisher Scientific Inc., USA) and incubated with total cell lysates of RL2-treated MDA-MB-231 cells. Control samples were incubated with total cell lysates of RL2-treated pancreatic cancer cell line SUIT-020. The precipitates were analyzed by nano-LC-tandem mass spectrometry and Western Blot.

Sample preparation for mass spectrometry was performed* via* on-bead digestion [24]. In brief, beads were rehydrated in 50 mM NH_4_HCO_3_, pH 8.0, and subsequently incubated with 1 mM DTT at 56°C for 45 min. Afterwards, reduced cysteine residues were ß-methylthiolated* via* the addition of 5 mM methyl methanethiosulfonate at room temperature for 30 min. Proteins were digested by adding 0.5 *μ*g trypsin (Trypsin Gold, Promega) and incubated at 37°C overnight. The generated tryptic peptides were eluted from the beads with two washing steps using 50 *μ*l of 25 mM NH_4_HCO_3_ for each wash. The washes corresponding to one sample were pooled together and dried in a vacuum centrifuge. The peptides were dissolved in 5 *μ*l 0.1% trifluoroacetic acid (TFA) and purified on ZIP-TIP, C18-nanocolumns (Millipore, Billerica, USA). The peptides were eluted in 7 *μ*l of 70% (v/v) ACN and subsequently dried in a vacuum centrifuge. LC-MS/MS was performed on a hybrid dual pressure linear ion Trap/Orbitrap mass spectrometer (LTQ Orbitrap Velos Pro, Thermo Scientific, San Jose, CA) equipped with an EASY-nLC Ultra HPLC (Thermo Scientific, San Jose, CA). The peptide samples were dissolved in 10 *μ*l of 2% acetonitrile (ACN)/0.1% trifluoroacetic acid (TFA) and fractionated on a 75 *μ*m I.D., 25 cm PepMap C18-column, packed with 2 *μ*m resin (Dionex, Germany). Separation was achieved by applying a gradient from 2% ACN to 35% ACN in 0.1% formic acid (FA) over a 150 min gradient at a flow rate of 300 nl/min. The LTQ Orbitrap Velos Pro MS exclusively used CID-fragmentation when acquiring MS/MS spectra consisting of an Orbitrap full MS scan followed by up to 15 LTQ MS/MS experiments (TOP15) on the most abundant ions detected in the full MS scan. The essential MS settings were as follows: full MS (FTMS; resolution 60.000; m/z range 400–2000); MS/MS (Linear Trap; minimum signal threshold 500; isolation width 2 Da; dynamic exclusion time setting 30 s; singly charged ions were excluded from selection). Normalized collision energy was set to 35%, and the activation time was set to 10 ms. Raw data processing and protein identification of the high resolution Orbitrap data sets were performed with* de novo* sequencing algorithms of PEAKS Studio 8.0 (Bioinformatics Solutions). The false discovery rate was set to <1%.

#### 2.2.9. Mouse Experiment

All procedures involving animals were performed in compliance with the protocols and recommendations for proper use and care of laboratory animals (ECC Directive 86/609/EEC). The protocol was approved by the Committee on the Ethics of Animal Experiments of the Administration of the Siberian Branch of the Russian Academy of Science (# 40 from April, 4, 2018). Female 8-9-week-old CBA mice were intramuscularly inoculated in right leg with RLS cells. Tumor volumes were monitored every 2 days. On the 8th day after the tumor transplantation, RL2 (12 mg/kg) was injected intravenously via the tail vein and such injections were repeated every 2-3 days with a total of 4 injections. CQ (50 mg/kg) was injected intraperitoneally daily with a total of 10 injections. When tumors reached an average volume of 50 mm^3^, animals were divided into groups. Control mice received i.v. saline (0.2 mL, 4 injections each 2-3 days). After the first injection, tumor volume and mouse weight were measured twice a week.

#### 2.2.10. Statistical Analysis

The Mann-Whitney U-test was used to define statistically significant differences for* in vitro* experiments. Student's t-test was used in* in vivo* experiments for comparing between two groups. Results are reported as the mean ± standard deviation (SD). P value less than or equal to 0.05 was considered as significant.

## 3. Results

### 3.1. RL2 Activates Concentration-Dependent ROS Production in Treated Cells

Earlier we demonstrated that lactaptin analog RL2 induces apoptotic markers such as caspase-3/-7 activation and mitochondrial membrane depolarization in cancer cells [14, 25].

**Figure 2:**
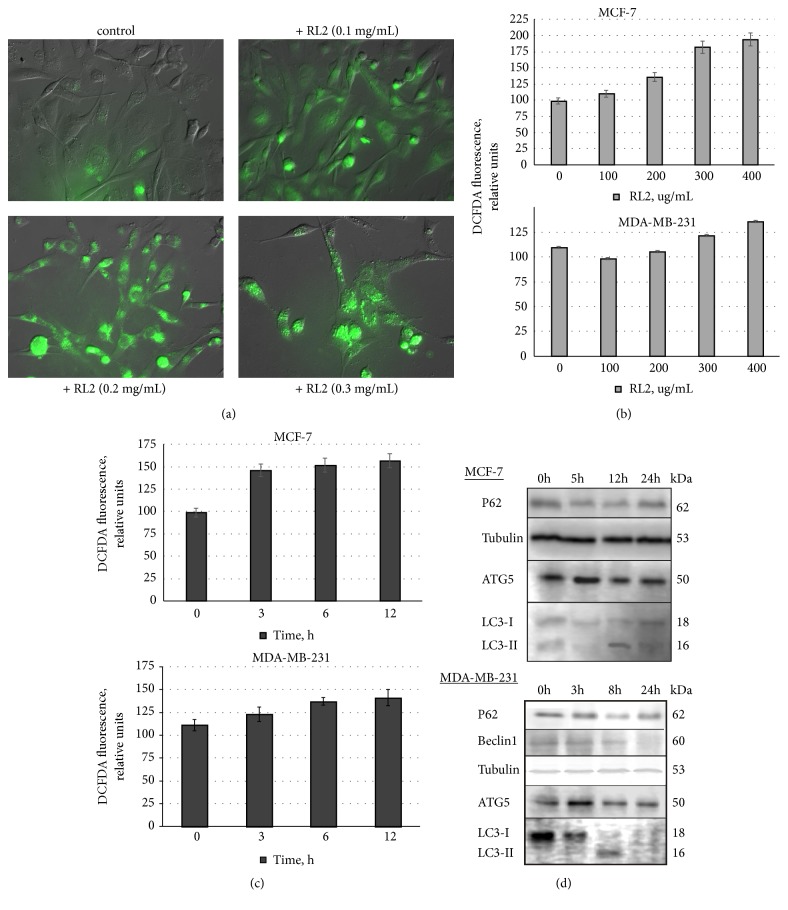
Analysis of ROS production and changes in autophagy-related proteins. (a) Representative fluorescence microscopy images of ROS production (green) in RL2-treated MDA-MB-231 cells. (b), (c) Dependence of ROS production on RL2 concentration and time of incubation. For a kinetic study of ROS production, cells were treated with RL2 (300 *μ*g/mL). (d) The representative Western Blot of MCF-7 and MDA-MB-231 cells treated with RL2 (300 *μ*g/mL) is showing a level of autophagy-related proteins.

Besides classical apoptotic pathways, excessive reactive oxygen species (ROS) can lead to cell damage and apoptosis. Using the ROS-sensitive fluorescent probes DCFDA (detecting mainly H_2_O_2_) and DHE (O_2_-specific) to monitor cellular oxidative stress, we found that RL2 increased the production of ROS in MDA-MB-231 and MCF-7 cells in concentration-dependent manner, but not in a time-dependent manner (see Figures 2(a)–2(c)). The increase of ROS production was well-detected after 3 h and it stayed high with no statistically valid changes during 12 h of incubation.

### 3.2. RL2 Stimulates Changes of Autophagy-Related Proteins

RL2 effects on autophagy-related proteins were analyzed. We observed a reduction of p62 levels in MDA-MB-231 cells after 8 h of incubation (see Figure 2(d)). Autophagy-related LC3-II form was detected at 3 h and 8 h of incubation, though its levels decreased by 24 h. The latter can be due to the proteolytic degradation of LC3II. We observed that Beclin 1 started to decrease from 8 h of incubation with RL2. ATG5 level was slightly higher in treated cells than in control cells. To summarize, RL2 activates autophagy-like molecular changes in MDA-MB-231 cells, which were most prominently observed at 8 h after treatment with RL2.

### 3.3. CQ Increases Cytotoxicity of RL2-Treated Cells and Changes Autophagy and Apoptosis Features

**Figure 3:**
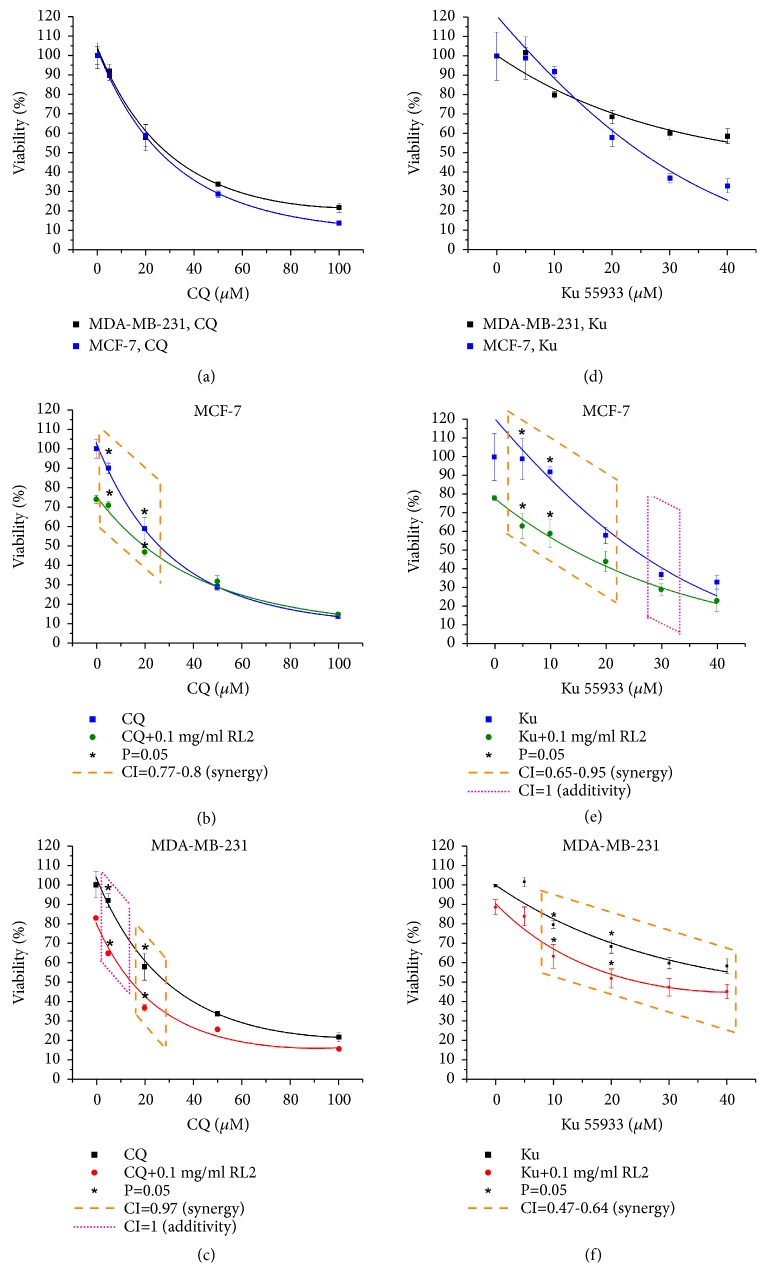
The influence of CQ and Ku with RL2 on viability of MDA-MB-231 cells and MCF-7 cells. Detection of viability was produced by MTT assay. (a)–(c) Cytotoxic effect of CQ (0-100 *μ*M) alone and in combination with RL2 (0.1 mg/mL) on MDA-MB-231 and MCF-7 cells. Combinatorial index (CI) is 0.77-1. (d)–(f) Cytotoxic effect of Ku (0-40 *μ*M) alone and in combination with RL2 (0.1 mg/mL) on MDA-MB-231 and MCF-7 cells. Combinatorial index (CI) is 0.47-1. CI was calculated with CompuSyn version 1.0.

MDA-MB-231 and MCF-7 cells were incubated with CQ alone and in combination with RL2 for 48 hours, and then cytotoxic effects were measured by MTT assay (see Figures 3(a)–3(c)).

We varied CQ concentration from 0.5 *μ*M to 100 *μ*M while RL2 concentration was constant. The dose-dependent decrease of viability of MDA-MB-231 and MCF-7 cells, treated with CQ, was observed. CQ enhanced the cytotoxic effect of RL2 in MDA-MB-231 and MCF-7 cells compared with CQ alone (see Figures 3(b) and 3(c)). These differences were more substantial if CQ concentration was used in the noncytotoxic range (0.5-20 *μ*M interval). The combinatorial index (CI) for MDA-MB-231 cells was 0.97 and for MCF-7 cells was 0.77; this indicates a synergistic effect of CQ and RL2.

**Figure 4:**
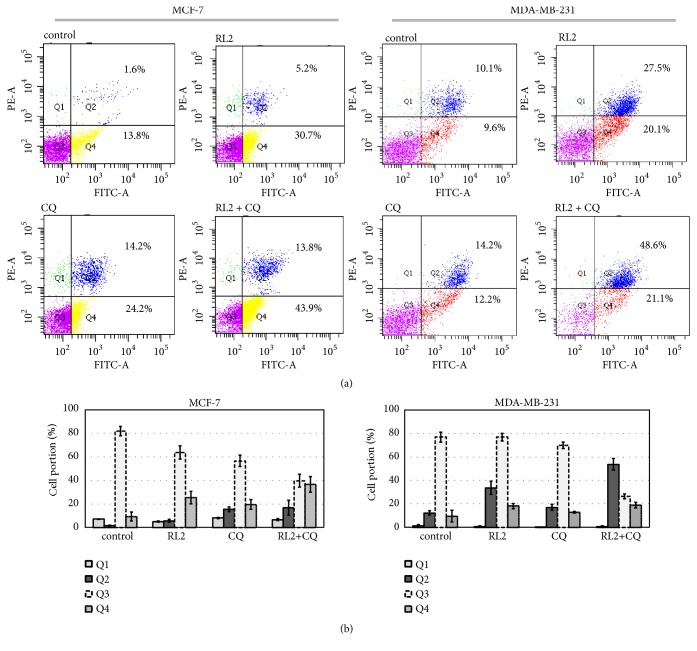
RL2 in combination with CQ increases percentage of dead cells. MCF-7 and MDA-MB-231 were treated with RL2 (0.15 mg/mL), CQ (20 *μ*M), or both compounds. After 24 h of incubation, cells were collected, stained with FITC Annexin V Apoptosis detection kit (BD), and analyzed with flow cytometry. (a) The typical cell distributions in Q1-Q4 quadrants. (b) The histograms show typical cell distributions of three independent experiments.

Next, we analyzed whether a combination of CQ with RL2 activates apoptosis in treated cells. MDA-MB-231 and MCF-7 cells were treated with RL2, CQ alone, and the combination. After 24 h of incubation, cells were stained with Annexin V/PI and analyzed by flow cytometry. We showed that Annexin V+/PI+ (Q2, late apoptotic) populations in MDA-MB-231 cells and Annexin V+/PI- (Q4, early apoptotic) populations in MCF-7 cells were significantly higher after combinatorial treatment in comparing (see Figure 4). As we observed, the live cells population was the lowest in combinatorial treatment and this is in good correlation with MTT data.

**Figure 5:**
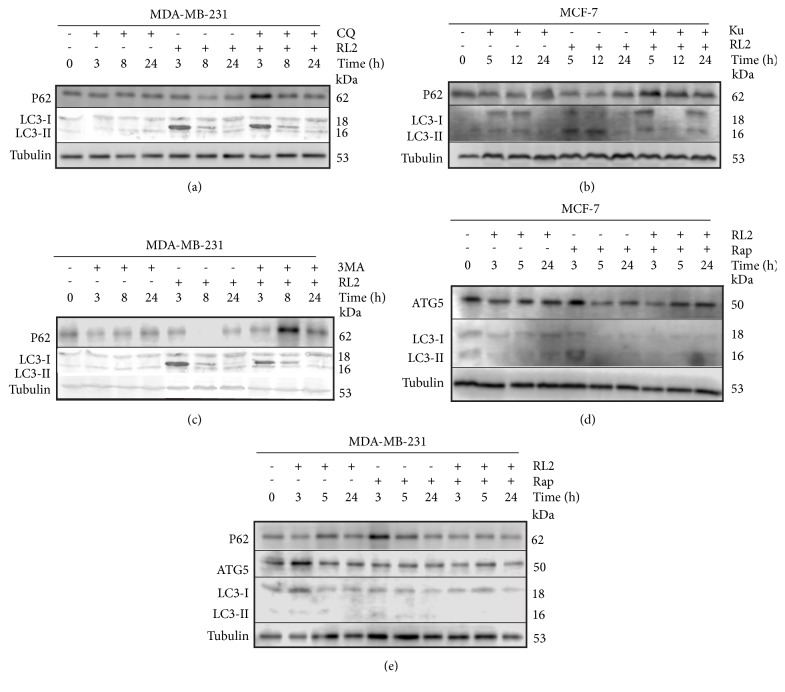
Changes in the cellular proteins after their incubation with indicated compounds. Representative Western Blots showing changes in autophagy-related proteins. Whole cell lysates were prepared for analysis using tubulin as a loading control. (a), (c), (e) Western Blots with MDA-MB-231 cell lysates. Cells were treated with RL2 (0.3 mg/mL), CQ (10 *μ*M), 3MA (10 mM), and Rap (10 *μ*M) for various time points (0–24h). (b), (d) Western Blots with MCF-7 cell lysates. Cells were treated with RL2 (0.3 mg/mL), Ku (30 *μ*M), and Rap (10 *μ*M) for the indicated time points (0–24h).

Next, we examined autophagy markers in MDA-MB-231 cells, treated with RL2 or/and CQ by immunoblot assay. This combinatorial treatment led to the accumulation of p62 protein with a maximum amount occurring at 3 hours in the treatment of MDA-MB-231 cells (see Figure 5(a)). The increase of p62 could indicate the autophagy suppression. Thus, CQ in combination with RL2 effectively decreased the viability of MDA-MB-231 and MCF-7 cells via inhibiting autophagy and amplifying cell death.

### 3.4. Combination of RL2 with Ku55933 Increases Cytotoxic Effect

Ku55933 (Ku), an ATM specific inhibitor, was also demonstrated to inhibit autophagy [20]. Ku decreased viability of MCF-7 cell more effectively compared to MDA-MB-231 with inhibitory concentration 50 (IC50) values of 26.1 *μ*M and 57.2 *μ*M, respectively (see Figures 3(d)–3(f)). Strong synergistic effects with RL2 were observed when Ku was used up to IC50 concentrations (see Figures 3(e) and 3(f)). The highest increase of cytotoxicity of such combination was observed with the lowest concentrations of Ku in the MCF-7 cells. The combinatorial index for 1-20 *μ*M Ku with RL2 (0.1 mg/ml) was 0.65, which indicates synergistic effects. Despite the fact that the cytotoxicity of RL2 with Ku was lower for MDA-MB-231 cells than for MCF-7 cells, a high synergistic effect (combinatorial index CI=0.47-0.64) was observed for these compounds in MDA-MB-231 cells.

Because MCF-7 cells were more sensitive to Ku than MDA-MB-231 cells and also more sensitive to the combination of Ku with RL2, the autophagy-related proteins p62 and LC3 were analyzed in treated MCF-7 cells (see Figure 5(b)). We revealed that RL2 (0.3 mg/ml) in combination with Ku (30 *μ*M) led to the accumulation of p62 when compared to control nontreated cells which indicates autophagy suppression. Ku alone slightly induced LC3II formation. Compared to RL2-treated cells, LC3I to LC3II processing was less strong in samples treated with the combination of Ku and RL2 (see Figure 5(b)). We concluded that this finding confirmed autophagy-suppressive action of Ku.

### 3.5. 3-Methyladenine (3MA) Does Not Increase Cytotoxicity of RL2-Treated Cells

**Figure 6:**
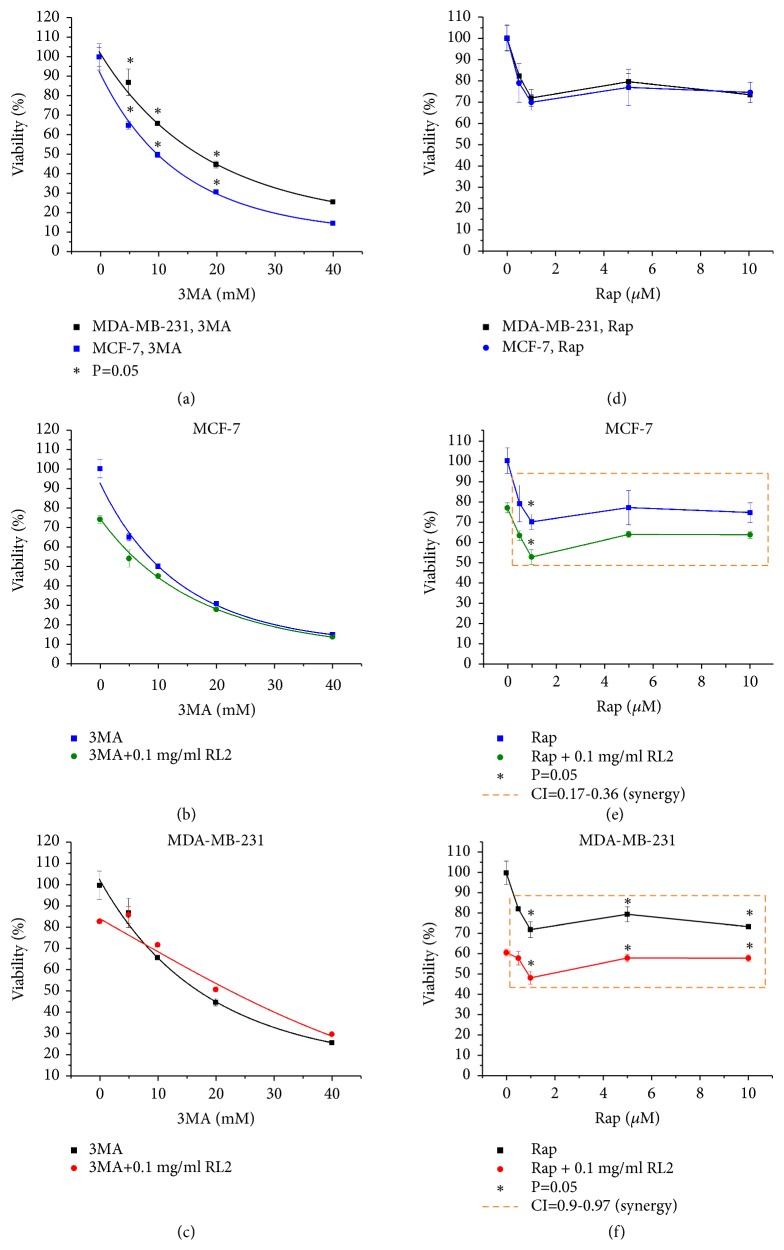
Combination of 3MA and Rap with RL2 influence on viability of MDA-MB-231 cells and MCF-7 cells. Detection of viability was produced by MTT assay. (a)–(c) Cytotoxic effect of 3MA (0–40 mM) alone and in combination with RL2 (0.1 mg/mL) on MDA-MB-231 and MCF-7 cells. (d)–(f) Cytotoxic effect of Rap (0-10 *μ*M) alone and in combination with RL2 (0.1 mg/mL) on MDA-MB-231 and MCF-7 cells. Combinatorial index (CI) is 0.17-0.97. CI was calculated with CompuSyn version 1.0.

The cytotoxic effect of RL2 (0.1 mg/ml) in combination with 3MA (0-40 mM) was investigated on MCF-7 and MDA-MB-231 cells. We observed no increase of cytotoxic effect in the combination of 3MA and RL2 in both MCF-7 and MDA-MB-231 cells when compared to monotreatment (see Figures 6(a)–6(c)). Increase of p62 levels appeared when cells were treated with RL2 and 3MA (10 mM) (see Figure 5(c)). LC3 analysis revealed that combinatorial treatment of 3MA and RL2 led to less LC3II than RL2 alone indicating a decrease of autophagy level.

### 3.6. Cytotoxicity of RL2 in Combination with Autophagy Inducers Rapamycin (Rap) and Spermidine

To examine whether rapamycin increases or decreases viability of RL2-treated cells, we incubated MDA-MB-231 cells and MCF7 cells with a combination of drugs and performed MTT analysis. Galluzzi et al. state that rapamycin concentrations higher than 10 *μ*M are likely to promote acute cell death mainly through off-target mechanisms [26]. Keeping this restriction in mind, we limited Rap concentration to 1-10 *μ*M. We revealed that both cell lines were low sensitive to rapamycin (see Figures 6(d)–6(f)). Under combinatorial treatment, synergistic effects were observed for the full range of Rap concentrations used (see Figures 6(e) and 6(f)). Performing Western Blot analysis, we observed that the combination of RL2 with Rap decreased p62 expression (see Figure 5(d)).

The disappearance of LC3II accompanied by the decrease of LC3I could be a consequence of proteasomal degradation of LC3 during autophagy which was initiated by both compounds. Under combinatorial treatment, a decrease in ATG5 levels was also observed (see Figures 5(d) and 5(e)). Taken together, a high level of autophagy induced by RL2 and Rap led to cell death.

Besides Rap, another autophagy inducer, spermidine, was analyzed in combination with RL2. We revealed that RL2 did not increase cytotoxicity of spermidine.

### 3.7. Autophagy Modulators Change Cathepsin D Activity in RL2-Treated Cells

**Figure 7:**
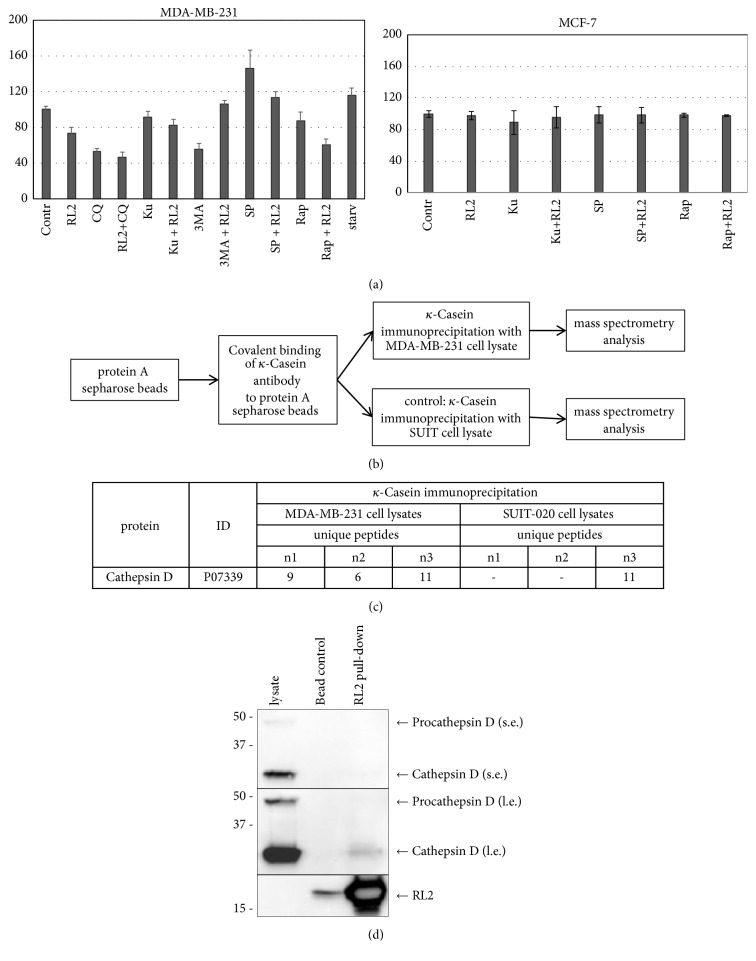
Cathepsin D effects. (a) Changes in CatD activity. MDA-MB-231 and MCF-7 cells were treated with RL2 (0.2 mg/mL), CQ (5*μ*M), Ku (30 *μ*M), 3MA (15 mM), spermidine (6 *μ*M), and Rap (15 *μ*M) for 24 h, and after that CatD activity in lysates was measured using fluorescent substrate. Starvation (starv) was initiated by serum deprivation. The data are presented as percentage from control (nontreated cells) and shown as the mean ± SD of three independent experiments. (b) Workflow for RL2-based mass spectrometry analysis. *κ*-Casein antibody protein A sepharose beads were loaded with *κ*-Casein antibody and incubated with RL2-treated MDA-MB-231 cell lysates or RL2-treated SUIT-020 cell lysates (control). Precipitated proteins were identified by nano-LC-tandem mass spectrometry. (c) Mass spectrometry identified unique peptides for each protein in the course of identification. The absolute values of unique peptides for Cathepsin D identified by mass spectrometry analysis are shown here. The abbreviations n1, n2, and n3 indicate absolute values for every experimental replicate. (d) RL2-pull-down precipitates were analyzed by Western Blot and probed for indicated proteins (bead con: control IP without antibody).

Earlier we have demonstrated RL2 decreased cellular Cathepsin D (CatD) activity in MDA-MB-231 cells when used in combination with CQ [14]. RL2 slightly decreased CatD activity whereas CQ decreased up to 2-fold. Testing here other autophagy modulators, we showed that CatD activity in cells treated with combination of RL2 and Ku or 3MA was not changed compared to control cells or RL2-treated cells (see Figure 7).

Next we investigated if autophagy inducers, Rap and spermidine, modulate CatD activity. We observed that Rap and spermidine have opposite effects on CatD activity: a valid decrease of its activity in Rap-treated cells was detected while in spermidine-treated cells its activity was increased. Interestingly, the addition of RL2 normalized (decreased) CatD activity in spermidine-treated cells to control cell's activity. The addition of RL2 to Rap-treated cells slightly decreased CatD activity compared with mono-Rap treatment. Taken together, the combinatorial treatment with RL2 and Rap or with spermidine showed a decrease of Cathepsin D activity. Importantly, we can show the interaction between CatD and RL2 by both mass spectrometry and Western Blot (see Figures 7(b) and 7(d)) suggesting that RL2 might trigger CatD activity via direct interactions with CatD.

### 3.8. Autophagy Modulators Decrease ATP Release from RL2-Treated Cells

**Figure 8:**
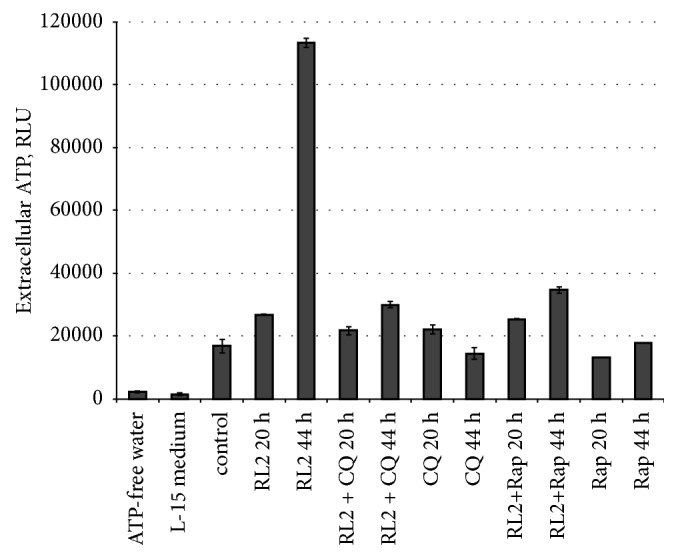
Analysis of drug-related ATP release. MDA-MB-231 cells were treated with RL2 (0.2 mg/mL), CQ (5*μ*M), and Rap (15 *μ*M) for 20 or 44 h; after that medium was collected and tested with ATP-specific luminescent substrate. RLU: relative luminescence units. The data are representative of three independent repeats and are shown as the mean ± SD.

Cell death associated with autophagy can also display signals of immunogenic death [1]. It was stated that autophagy is required for chemotherapeutics to induce ATP release. We investigated if RL2, CQ, Rap, or their combinations activate ATP release from treated cells. We observed that ATP release from RL2-incubated cells was about 10 times higher than that from nontreated cells (see Figure 8). Chloroquine and rapamycin did not change ATP release compared to nontreated cells. When we incubated cells with a combination of these compounds with RL2, the amount of external ATP was slightly higher than that for samples incubated only with CQ or Rap, but it was substantially lower than that for RL2 alone. This indicates that CQ and Rap prevent massive ATP release from dying cells incubated with RL2.

### 3.9. Estimation of Autophagosome/Autophagolysosome Formation in Treated Cells

**Figure 9:**
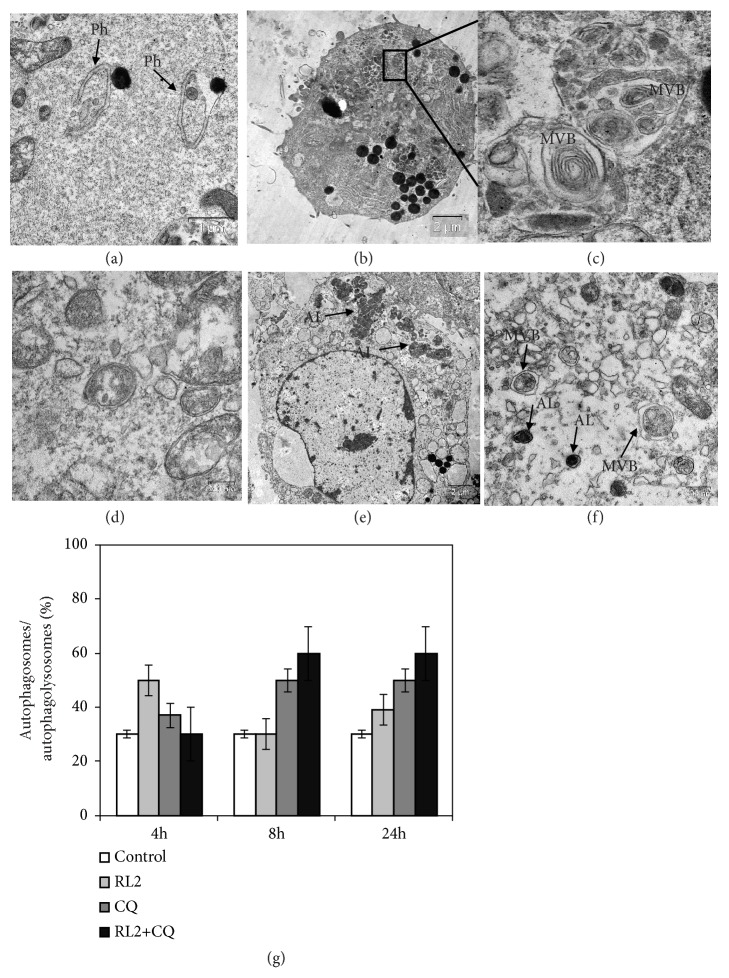
RL2 and autophagy modulators affect autophagosome/autophagolysosome formation in MDA-MB-231 cells. (a)–(f) Electron microscopy images of cross-sections of cells treated with RL2 (0.3 mg/mL), CQ (20*μ*M), and Rap (100 nM). (a) phagophore formation in RL2-treated cells. (b), (c) Fragment of RL2-treated cell with multiple autolysosomes. (d) Autophagosomes in Rap-treated cells (8h). (e) Dead cell after Rap+RL2 treatment (24 h). (f) Autophagosomes in a dead cell after CQ+RL2 treatment (24 h). Asterisks indicate phagophore (Ph), multivesicular body (MVB), or autolysosome (AL). (g) Percentage of autophagosomes/autophagolysosomes in MDA-MB-231 cells treated with RL2 and CQ.

For direct visualization of autophagy, we analyzed cellular ultrastructure by the electron microscopy (see Figures 9(a)–9(f)). After 4 h of incubation, only cells treated with RL2 alone showed an elevated amount of autophagosomes/autophagolysosomes (see Figure 9(g)). Cells treated with RL2 and CQ for 8-24 h contained a twofold increase of autophagosomes compared to control cells or RL2-treated cells. CQ alone also led to an increase of autophagosomes/autophagolysosomes after 8-24 h of incubation. These data conform with the fact that CQ prevents productive autophagy with complete catabolic processes that lead to the accumulation of autophagosome into the cells.

### 3.10. RL2 in Combination with CQ Suppresses RLS Allograft Growth

**Figure 10:**
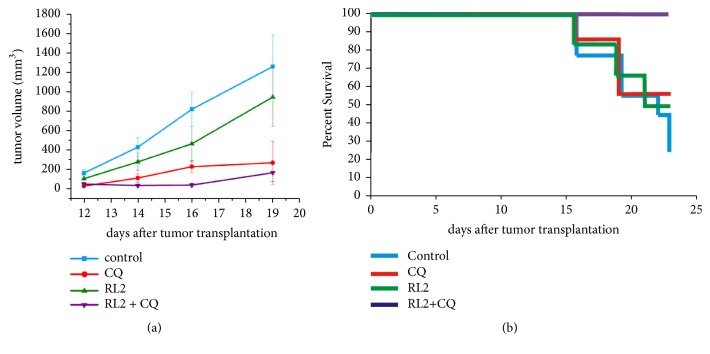
RL2 in combination with CQ delays tumor development. Female CBA mice aged 8-9 weeks were used for intramuscular RLS cell transplantation. The mice were randomly divided into 4 groups (6-9 mice in group): RL2 (12 mg/kg) dissolved in saline was administered i.v. via the tail vein every 2-3 days starting on day 8 after the tumor transplantation, CQ (50 mg/kg) dissolved in saline was administered i.p. daily, RL2 in combination with CQ was administered as described above, respectively. (a) The growth rate of RLS tumors. (b) Lifespan of control and experimental mice. Error bars represent mean ± standard deviation. The statistical difference in median tumor volume between the control and the experimental groups was *∗*P<0.05. Student's t-test was used for statistical analysis. Results are reported as the mean ± standard deviation (SD).

To test whether RL2 in combination with CQ delays tumor growth* in vivo* more efficiently than RL2 and CQ alone, a mouse allograft model of cyclophosphamide-resistant lymphosarcoma RLS was used. A suspension of RLS cells was intramuscularly injected into the mice's right leg to induce tumor development. Animals received RL2 (12 mg/kg) i.v. and/or CQ (50 mg/kg) i.p. while control tumor-bearing mice received NaCl i.p. We showed that RL2 in combination with CQ significantly delayed tumor growth compared to the control group (p<0.025). There were no significant differences between the CQ-treated group and the RL2+CQ-treated group (p>0.1) (see Figure 10(a)). The tumor growth inhibition rate was calculated as 86.5% (group RL2 and CQ), 24.9% (group RL2), and 78.4% (group CQ). Another supervised characteristic was the lifespan of the tumor-bearing mice (see Figure 10(b)). By day 23 after the tumor transplantation, all animals which received RL2 and CQ were alive. The percentage of survival for other groups was lower: 57.1% in CQ-receiving group, 50% in RL2-treated group, and 22.2% in control group. By this date the comparison of tumor sizes between the groups was nonnegligible due to the death of a major part of the animals in the control group.

## 4. Discussion

Nowadays, the fundamental value of autophagy in living organisms is obvious. Autophagy enables cells to adapt to stressful conditions including cancer cell survival under hypoxic and low-nutrient conditions. Its role in cancer progression and chemoresistance makes autophagy an appropriate target for anticancer therapy [27, 28]. Meanwhile, several conventional chemotherapeutics induce cancer cell death through autophagy-related mechanisms [29]. Here we investigated whether analog of proapoptotic protein lactaptin (RL2) activates autophagy-related pathways in cancer cells and whether autophagy modulators improve cytotoxic activity of RL2. Autophagic cell death (type II programmed cell death) is primarily independent of the activation of caspases and morphologically different from apoptosis [17]. In comparison with apoptosis, autophagic cell death is characterized by the massive accumulation of autophagosomes, limited chromatin condensation, and lysosomal protein LC3 maturation.

In the current study we have demonstrated that RL2 induced a short-term autophagy at the initial stage of incubation* in vitro*. Western Blot analysis revealed changes of the major autophagy-related proteins in RL2-treated cells. Electron microscopy confirmed autophagosome formation which was induced by RL2. Moreover, we have detected that RL2 stimulated dose-dependent ROS production. Excessive cellular levels of ROS can cause damage to molecules and organelles, such as mitochondria [30]. For example, H_2_O_2_ causes initial mitochondrial membrane potential (MMP) hyperpolarization leading to the decrease of MMP, mitochondrial translocation of Bax and Bad, and cytochrome c release [31]. In conjunction with our previous data on the disruption of the inner mitochondrial membrane potential following the RL2 treatment, we suggested that ROS can be engaged in RL2 cytotoxicity.

Chemicals which activate or deactivate autophagy pathways can govern therapeutic-dependent death of cancer cells. We investigated cytotoxic effects of RL2 in combination with different autophagy inhibitors and inducers on MDA-MB-231 and MCF-7 cells. We have used well-described specific autophagy inhibitors (CQ, 3MA) and inducer (Rap) as well as nonspecific autophagy modulators like inhibitor Ku and inducer spermidine. We have shown that only CQ, Ku, and Rap synergized prodeath activity of RL2. It was not clear why autophagy activator Rap became so effective in combination with RL2 to be an autophagy inhibitor. When we used CQ with RL2, the increase of cytotoxicity was accomplished by effective suppression of RL2-induced autophagy according to Western Blot analysis. However, electron microscopy showed higher amounts of autophagosomes in CQ-treated MDA-MB-231 cells compared with nontreated cells. CQ-dependent accumulation of autophagosomes is well described and originates from the prevention of the degradation process [32, 33]. Massive accumulation of autophagosomes is one of crucial characteristics of autophagic cell death [34, 35]. An analysis of the phosphatidylserine externalization in cells treated with CQ and RL2 by flow cytometry showed that the decrease of living cells was accomplished by the substantial increase of secondary apoptotic (Annexin V+/PI+) cells but not early apoptotic (Annexin V+/PI-) cells compared to RL2-treated cells (see Figure 4). These data indicate that the combination of CQ and RL2 amplified cell death and that this is diverse from apoptosis and is more likely autophagic cell death. In comparison with CQ, 3MA blocks the early stage of autophagy. It can inhibit the formation of Beclin 1-PI3KC3 complexes as well as conversion of soluble LC3-I to lipid bound LC3-II and the formation of autophagosomes. We suppose that the suppression of autophagosome formation by 3MA excludes the possibility of autophagic cell death which apparently takes place in CQ/RL2-treated cells.

Autophagy inducer rapamycin is a macrolide immunosuppressant which is licensed for use against solid organ and bone marrow rejection [36]. Rap inhibits immune cell growth and proliferation by blocking signal transduction [37]. Usually the amount of LC3II correlates with the amount of autophagosomes and we expected that Rap could intensify RL2-dependent autophagy. When we used Rap with RL2, we observed that Rap efficiently induced LC3I to LC3II (lipidation) processing at an early stage of incubation (3-5 h), but when subjected to longer incubation, both LC3I and LC3II disappeared (see Figures 5(d) and 5(e)). This is because under Rap-initiated autophagy, LC3II is also degraded, which correlates with other investigators [38].

We found that p62 was sensitive to combination of RL2 with all autophagy inhibitors used, CQ, Ku, and 3MA, but only CQ and Ku synergized cytotoxic effect with RL2. Hence, autophagy inhibition is not sufficient for the increase of cytotoxicity. The amounts of LC3II and p62 are reliable markers of autophagy but only the morphological assessment with electron microscopy can confirm autophagosome involvement. The increased number of autophagic vesicles which were observed in the cells incubated with combinations of Rap/RL2 or CQ/RL2 may be a result of autophagy induction for Rap and its blockade at downstream steps for CQ [18]. In both cases the accumulation of autophagosomes led to a significant increase in cell death.

The activity of lysosomal proteases produces autophagic protein degradation. Cathepsin D is a lysosomal aspartyl protease involved in the degradation of unfolded, damaged, and unused proteins. Alteration of CatD enzymatic activity leads to changes in modulation of diverse enzymes and growth factors which are crucial in various proteinopathies and cancer [39–41]. It is known that lysosomal cathepsins are involved in apoptotic and nonapoptotic cell death [42]. The release of CatD increases the release of cytochrome C and subsequently activates caspase-3/-7, thus inducing the depolarization of the mitochondrial membrane, linking defective autophagy to caspase-dependent apoptosis [41]. Alternatively, cytoprotective activity of CatD against excessive aggregated proteins was demonstrated as well, and this activity was independent of autophagy [43]. We consider that when entering into the cells, RL2 can stimulate proteases, in particular CatD, responsible for the degradation of excessive and unused proteins. However, we revealed a decrease of CatD activity in the cells incubated with RL2. This finding indicates that RL2 is more likely to act as CatD suppressor and that this can be realized through their direct physical interaction. Among autophagy modulators used in this study spermidine and 3MA prevent a RL2-dependent decrease of CatD activity while CQ and Rap intensify CatD deactivation. We suppose that the decrease of CatD enzymatic activity is essential for high cytotoxic activity of the combination of RL2 with CQ or Rap. In our body there is an endogenous pool of spermidine with no toxicity under physiological concentration. An exogenous supply of spermidine can induce macroautophagy with increased autophagic flux by inhibiting the acetyltransferase EP300 [21]. By this pathway, spermidine reduces global protein acetylation of cellular proteins that can lead to CatD activation [44]. According to our data, spermidine acts as CatD activator and it was the least potent for increase of cytotoxic activity of RL2.

Chemotherapy-induced release of ATP from dying cells leads to the increase of extracellular ATP acting as a “find me” signal for the recruitment of phagocytes. Autophagy contributes to this phagocytic targeting of apoptotic cancer cells. Moreover, dying autophagic cells can release ATP [45]. In our experiments, a treatment of MDA-MB-231 cells with RL2 resulted in a fivefold increase of extracellular ATP; this could be indirect evidence of autophagy activation. When we coincubated the cells with RL2 and autophagy modulators, we observed that CQ and Rap prevent massive ATP release from dying cells. This is in line with other studies where it was demonstrated that the suppression of autophagy inhibited the release of adenosine triphosphate from chemotherapy-treated tumor cells [1]. It was also demonstrated that inhibition of autophagy with Rap failed to trigger ATP release. Therefore, in spite of the high cytotoxicity of the combination of RL2 and CQ, it is more expected that cells after such combinatorial treatment will fail to prime T cells* in vivo *and to activate antitumor immunity.

We have also tried to translate our* in vitro* results to an animal model. Antitumor potential of CQ with various efficiencies has been demonstrated in several works [46–48]. This compound has a low toxicity itself and can be injected intraperitoneally into mice, which makes CQ therapy of a mice cancer model a very suitable investigation of combinatorial treatment. We showed that the treatment of the tumor-bearing mice with RL2 and CQ improved the antitumor effect of RL2. Taking into account the high speed of tumor progression and the mortality rate of the mice of a nontreated group, we did not observe statistically valid differences between CQ-treated and CQ-with-RL2-treated animals. However, the viability rate of animals receiving RL2 and CQ was significantly higher than that in animals treated with CQ alone.

## 5. Conclusions

This study provides new insights into the mechanisms involved in recombinant lactaptin RL2 cytotoxic activity. Based on our results, we suggest that the combination of RL2 with chloroquine is a promising antitumor approach against breast cancers.

## Data Availability

All data used to support the findings of this study are available from the corresponding author upon request.
